# Resilience Among Language Learners: The Roles of Support, Self-Efficacy, and Buoyancy

**DOI:** 10.3389/fpsyg.2022.854522

**Published:** 2022-03-11

**Authors:** Wenjiao Li

**Affiliations:** ^1^School of Marxism, Southwestern University of Finance and Economics, Chengdu, China; ^2^Polus International College, Chengdu, China

**Keywords:** buoyancy, language learners, resilience, self-efficacy, social support

## Abstract

The growth of positive psychology has generated different perceptions and concepts on the authorization of learners, such as the construct of resilience and buoyancy. It has been argued that buoyancy has a central function in the educational process as buoyant pupils are more about to participate in activities presented in the classroom and also they are interested to cope with stress in challenging situations especially in English as a foreign language (EFL) learning context. Moreover, to protect against these adversities in reactions to unexpected situations, a related concept exists in positive psychology, labeled resilience that designates persistence and underlines people’s capabilities in face of adversities. The expansion of factors such as self-efficacy and social support seem to have great impacts on different aspects of learners. To this end, the present review attempts to highlight these two noteworthy elements in managing learners’ resilience and buoyancy. Consistent with this review, some recommendations for future inquiries are presented and instructional implications are offered.

## Introduction

Based on the research, academic adversity, challenges, and threats increase the vulnerability of most learners to school dropout (Schultze-Lutter et al., 2016). Some of these difficulties can manifest themselves in the form of chronic academic failure, perceived academic stress, and inability to adapt to the academic and social setting, which often culminates when learners drop out of school (Schultze-Lutter et al., 2016). Indeed, schools and other areas of education are where academic problems, setbacks, and pressures are constant realities of daily life (Martin and Marsh, 2008). As a result, learners and teachers experience significant stress and mental health problems on the one hand (Jones, 2020) and on the other hand, it is difficult for some researchers to document the way educators deal with the factors affecting their learners’ problems (Anderson et al., 2020). Thanks to the advent of positive psychology (Wang et al., 2021), a significant section of the education is placed on the positive aspects such as a learners’ ability to be resilient and buoyant when facing academic risks and difficulties (Martin and Marsh, 2009). While numerous learners act poorly, many other learners can change their academic careers by overcoming initial problems and shortcomings. To overwhelm such threats and stresses of the educational environment, learners need some degree of resilience to continue. In line with Tudo and Spray (2017), resilience increases your chances of success in school and other life achievements despite adversity caused by your initial characteristics, conditions, and experiences. Over past years, resistance studies have become popular, and different models and data have come into existence, with criticism of the benefit of such construct in psychology. Resilience is described as global self-efficacy, which makes the construct measurable, thus, self-efficacy empowers individuals to handle troubles and stressors *via* getting aware of their own assets and sources (Lightsey, 2006). Based on previous research on resilience displays that resilience inclines to be dichotomized (Pidgeon and Keye, 2014). Martin and Marsh (2008) reported that the self-efficacy factor positively and predictively affects academic resilience, and learners indicate that learners with high resilience have high academic self-efficacy.

In order to protect students against educational difficulties and challenges, buoyancy is one of the elements that can be helpful that it talks about learners’ capabilities in dealing with these educational obstacles and hardships effectively (Martin and Marsh, 2020). Indeed, it involves developing individual powers by focusing on a proactive rather than reactive approach to learning failures and contests (Martin and Marsh, 2008). Buoyancy has been recognized as an issue that experts can develop to encourage learners positively to manage educational adversities as they appear in the process of learning (Collie et al., 2017). Buoyancy supports enthusiasm, enabling students to overcome the uncertainties of daily language learning, make sustained efforts and conquer problems on the route toward the realization of language learning (Kim and Kim, 2017).

Martin and Marsh (2009) anticipated both buoyancy and resilience as two significant issues that reinforce learners’ constructive relations to college and educational life and their capability to bounce back in case of dealing with different types of educational hardships. They have postulated that both constructs are not inherent skills—they can be enhanced in people through the advance of constructive intellectual, emotional and developmental orientations to school and educational life. Indeed, there are two psychological factors associated with resilience and buoyancy, risk factors and protective factors (Ballenger-Browning and Johnson, 2010). Risk factors are issues that develop the prospect of maladaptation, while protective factors are those features that can increase flexibility. Protective issues contain life fulfillment, hopefulness, constructive affectivity, self-efficacy, self-confidence, and social support.

Academic buoyancy is influenced by several issues, one of which is self-efficacy, explained as self-assurance in one’s capacity to perform academic assignments and reach academic goals (Ranjbar et al., 2019; Holloway-Friesen, 2021). Self-efficacy is a central concept of academic studies since there is a strong experimental link between convictions on self-efficacy, enthusiasm, and manner (Dörnyei and Ryan, 2015). Moreover, there are plentiful proofs to suggest that self-efficacy is one of the resilience components that have a significant function in the age of adversity and weakness (Fathi et al., 2020).

These convictions have a significant function in students’ emotions, thoughts, self-motivation, and the manner in scholastic environments (Schunk and Pajares, 2005). Self-efficacy is frequently referred to as semantic self-esteem in L2 education (Mills, 2014) and it is situation-dependent and appears to be especially significant when people encounter troubles and when constructive convictions on self-efficacy are related to heightened motivation and persistence as well as a heightened chance of refusing deconstructive notions about one’s own capacities (Bandura et al., 2001). Self-efficacy is the most basic human form of agency assisting people to cope with adversity and improve personal functioning and emotional well-being quality (Fathi et al., 2021). Similarly, self-efficacy enhances resilience by increasing competence and self-esteem in times of hard times (Hamill, 2003).

Alternatively, social support has been regarded as one of the most important elements in people’s endurance when conquering difficulties (Ronen et al., 2016). Social support acts as a shield vs. demanding experiences (Cluver et al., 2009), encouraging people to realize that they are loved, cared for, respected, valued, and in a mutual association (Rigby, 2000). Individuals frequently need others’ assistance to cope with learning and social difficulties and to have a sustainable and successful life, and these support factors are mentioned as factors of social support (Atnafu, 2012). People attain various knowledge utilizing social support. They attain knowledge that makes people have faith that others are fond of and worry about them, and knowledge that makes them have faith that they are valued and are part of a network of human relationships and reciprocated devotion (Agbaria and Bdier, 2020). Generally, perceived social support enables people to handle difficulties and recover from adversity (Mattanah et al., 2010).

Investigations reveal that social support from teachers, peers, and caregivers can enhance school performance in teenagers and prevent mental deterioration (Malecki and Demaray, 2006). Educators in class have the main function of relaying information, coaching, and training learners in their scholastic work. When contrasted with guardians and colleagues, educators had the most significant rank for offering instrumental assistance and knowledge support, as opposed to aspects like care, affinity, and respect (Wentzel, 2016). Nevertheless, with reference to the researcher’s knowledge, little is acknowledged about the role of efficacy and social support in the foreign education field and their noteworthy functions in augmenting resilience and buoyancy. Consequently, one of the notable purposes of the present review was to take the issue into account in EFL learning and research and examine their central functions on language learners’ resilience and buoyancy.

## Related Concepts

### Resilience

The concept “resilience” comes from the Latin verb “resilire,” which means to jump back (Windle, 2011; Fletcher and Sarkar, 2013) and it is generally characterized in glossaries as the capability of an individual to deal with difficulties, or the capability of a material or an item to return to its initial form after bending or stretching. According to a societally contextual perspective, resilience is a two-element notion always containing two related parallels: subjection to hardships and constructive adaptation to hardships (Johnston-Wilder and Lee, 2010). Undoubtedly, although the methods to characterize resilience are inconsistent, for resilience’s presence, the existence of two key notions is needed: hardships and constructive adjustment (Pooley and Cohen, 2010; Windle, 2011; Fletcher and Sarkar, 2013). As stated by Riley and Masten (2005), resilience is delineated as discussing forms of constructive adaptation in the case of hardship and define it as demanding that substantial difficulty or risk to adaptation or growth has happened and that operational is satisfactory, either due to the fact that satisfactory adaptation was achieved during the time of difficulty or due to the retrieval to satisfactory effective has been perceived. For some years, studies on resilience have regarded it as a dynamic cycle of intricate associations between a person and his or her sociocultural elements and have determined a set of risks and protective elements in the growth of students. Thus, it is crucial to determine what incorporates resilience to explain its notion. To try to study this notion, Earvolino-Ramirez (2007) argued that for a societally contextual notion to take place, precursors (occurrences that take place before the notion does) and results (occurrences that take place consequent to that of the notions) must exist. Hamill (2003) characterized resilience as ability in times of trouble, and Gilligan (2009) as a group of attributes that assist an individual to endure many of the deconstructive influences of difficulties. Resilience is characterized as the possibility of utilizing inner and outer sources to display creativity as a reaction to a variety of circumstantial and progressive difficulties (Pooley and Cohen, 2010; Xue, 2021).

Scholastically resilient learners are those sustaining great degrees of attainment, inspiration, and presentation despite the existence of distressing occurrences and states that put them at risk of performing unsuccessfully in school and, eventually, leaving school (Martin and Marsh, 2009). A review of the literature indicates that the concept of resilience is used in an extensive series of different domains, to express the perseverance of a system, a construction, or a public toward the disruption or disrupting fluctuations triggered by people or societal or ecological circumstances (Windle, 2011). With resilience, hardship is the major precursor, and constructive adjustment is the result.

There are two main methods of resilience research. Firstly, a factor-centric method that examines the relationship between the level of risk/misfortune and attributes that could keep an individual safe from deconstructive results and repercussions. Secondly, an individual-centric method that contrasts individuals with various outlines to ascribe what distinguishes resilient people from non-resilient ones. When contextualizing these methods to risk and resilience, one may also consider factor-centric methods to mainly include risk elements and individual-centric methods to mainly include at-risk people (Coleman and Hagell, 2007).

### Academic Buoyancy

Similar to scholastic resilience, another concept suggested by Martin and Marsh (2009) is scholastic buoyancy, which is the ability to conquer failures, difficulties, and hardships that are common in daily scholastic life (Martin, 2014). It is distinguished from scholastic resilience, which contrarily refers to the ability to conquer great misfortune that jeopardizes the academic growth of learners. There is proof that while buoyancy and resilience are connected, the former is better at indicating low-degree deconstructive results and the latter better indicates significant deconstructive results. This is in line with Martin and Marsh (2008) recent explanation of buoyancy as mirroring daily scholastic resilience. The term “buoyancy” was coined by Martin and Marsh (2008) as the aptitude to cope with everyday adversity and stressors and described it as daily resilience. They differentiated buoyancy from resilience in different routes. First, they said that both constructs vary in a simple description. Resilience refers to severe adversity, severe or prolonged, whereas buoyancy refers to problems about hindrances, stresses, and troubles in everyday life.

Theoretical buoyancy was regarded as a noteworthy interpreter of educational success and accomplishments, faculty satisfaction, class involvement, and overall self-confidence (Martin and Marsh, 2009; Martin, 2014). Learners who have a high degree of buoyancy similarly have high task accomplishment and commitment and a low level of non-attendance (Martin and Marsh, 2008). Moreover, buoyancy was related to other motivational upshots, like a high level of self-assurance, perseverance, and low level of apprehension (Martin et al., 2010).

### Self-Efficacy

Self-efficacy is the main issue in labeling how a person performs, feels, and reacts in face of opposing demanding situations and it is precarious in increasing learners’ presentation to make the scholastic development better (Van Dinther et al., 2011). Bandura (2011) believes that a central concept in social cognitive theory is self-efficacy which refers to the self-evaluation of people and their ability to perform the acts necessary to successfully achieve a specific goal. A beneficial apparatus for researchers in predicting persistence, emotional response, and effort is self-efficacy. In line with Martin and Marsh (2009), self-efficacy is a mental characteristic that students can improve. It is an important element in determining how a person behaves, thinks, and reacts in hard situations. It is essential to build students’ personalities to improve their learning cycle (Thompson and Verdino, 2019). Self-efficacy is a motivational factor in education, and it is rather impossible to explore some dimensions of individual roles like studying, motivation, and scholastic presentation, in spite of the function of students’ self-efficacy convictions. Self-efficacy in foreign language education has frequently been labeled as verbal confidence and it has been presented that a great level of efficacy is meaningfully related to success among EFL learners (Han and Wang, 2021).

When the belief in self-efficacy is stronger, the effort, perseverance, elasticity, and resilience will be greater. This means that a strong belief in self-efficacy generates the tranquility sense and empowers individuals in facing difficult tasks. It affects our thinking, emotions, deeds, motivation, and acts primarily *via* cognitive and emotional channels, and plays an important role in forming our perceived life experiences (Bandura, 2011). Bandura (2011) pinpointed that a person’s efficacy can be established *via* four foremost bases such as mastery experience, vicarious experience, vocal or societal influence, and stimulation or communicative state. Mastery experience is self-realized *via* temporary progressions, examining authorities for evidence and assistance, and personal knowledge or self-innovation education. Nevertheless, vicarious experience is the significance of looking at others carrying out a task. Verbal or social persuasion talks about the reassurance or opposition delivered by conservational social managers like managers, educators, parents, families, and classmates. The final resource refers to the state triggered by emotional and spiritual variables like enthusiasm, anxiety, and apprehension, which is opposed to an individual’s emotional state and influences their feelings. Students who enjoyed the high intellect of self-efficacy have been established to have better educational presentation along with being able of managing stress and other difficulties than those peers with a low level of efficacy (Feldman and Kubota, 2015).

### Social Support

The standard of the educator-learner connection is an important mechanism by which class experiences enhance learners’ condition and abilities in different aspects of growth (Chong et al., 2018). Educator support alludes to the degree to which learners have faith in their educators’ worth and are after creating individual connections with them to receive support and instrumental assistance to enhance their education and health (Ryan and Patrick, 2001). New meta-analyses have offered extensive proof of the influence of educators’ manners in supporting learners’ education and working across grade levels (Cornelius-White, 2007; Roorda et al., 2011).

Moreover, educator support has proved to be a further and separate contribution to emotional, behavioral, and intellectual involvement, where support from one area can make up for the absence of support from relatives and/or colleagues (Wentzel, 2016). The support provided by the teacher for learners’ education thus can disrupt a cycle through which learners start to present detaching manners and are a constructive resource for those experiencing support may be regarded as a cycle of enhancing societal relationships. It involves societal resources made available to people by a societal setting (Cohen et al., 2000). Support offered to educators has recently had a significant function in educator training studies especially emotional support that is referred to the educators’ comments (Bielak and Mystkowska-Wiertelak, 2020). The support educators obtain from relatives, school administrators, and peers have proved to be significant for expert progression (Skaalvik and Skaalvik, 2011; Song and Alpaslan, 2015).

Social support is deemed as having someone to propose support when such support is required and it can originate from the emotive background of family, friends, and peers (Hale et al., 2005). Also, it can be imminent thanks to cooperating with others’ social cycles, comprising experts and the milieu (Greenberger et al., 2000). Indeed, social support is a construct that embraces diverse proportions of perceived and functionally comprehended social argument, like gratification with the excellence and magnitude of support, feeling cared for, appreciated, and is related to an individual’s social system (Chronister et al., 2006). Minor social networks, fewer close relations, and lower competence of social support have been related to emotional distress and particularly depressive indications and mental condition (Kawachi and Berkman, 2001).

## Conclusion

This review could be supportive for scholastic psychologists, teachers, scholastic investigators, and material developers to establish some programs to improve the buoyancy and resilience degree of learners that has a great impact on learners’ presentation and learning level. Besides, this review might also offer societal and psychosomatic academics and learners with some aspects of personality and manners among those that need to be investigated later on. Evidence and ideas obtained from this review could assist learners to control and cope with their problems in the learning route.

It can be proposed that in preference to concentrating on eluding or decreasing the destructive aspects like pressure, anxiety, and tension that go along with education in general and language learning in particular, reinforcing constructive signs in the route of peripheral risk may positively strengthen students’ buoyancy and resilience in the procedure of language education, assist them to improve the aptitude to manage and cope with daily pressures and obstacles. To this end, generating and sustaining a great level of efficacy can be achieved through considering and improving learners’ principles about themselves and their capabilities in learning; purposefully rearranging education to take full advantage of the prospects to achieve; and imagining the achievement of similar people that empowers students to create influential objectives (Mills, 2014). Grounded on a wide range of literature reviews on resilience, it has been hypothesized that some school-based packages and treatments efficiently nurture resilience through constructing particular individual features such as buoyancy, self-efficacy, and problem-solving abilities.

On the whole, the review of the literature reveals that academic buoyancy signifies an indispensable yet under-examined capability that empowers students to be interested regardless of obstacles and experiences of troubles that establish actual learning upshots in educational language situations. The study of academic buoyancy and resilience is intended to clarify the capability of students to keep on interested, endure to be strong to obstacles and trials, and overwhelm the stresses that are part of the regular progress of education. People with high levels of resilience have sufficient self-confidence, good self-efficacy, and are capable of managing and solving issues. Furthermore, they are positive, pliable, and bounce back swiftly from distressing circumstances (Fredrickson, 2004). In addition, a robust sense of self-efficacy and perceived social support enhances people’s confidence, leading to positive health results, stimulating them to investigate potential interactions between these two variables regarding resilience (Karademas, 2006). Self-efficacy theories added to better academic buoyancy of learners accompanied by enlightening their homework, tasks, and class participation. As proposed in the theory of resilience, self-efficacy can prepare learners with interior affirms required to keep them away from educational difficulties and hardships. Along these lines, they are capable of increasing buoyancy that would permit them to control the difficult and demanding academic atmospheres directly and effectively.

People with high self-efficacy are more able to manage their thoughts and overcome difficulties when faced with adversity than those with low self-efficacy. In addition, it is known that the high self-efficacy of an individual is a major factor in predicting successful education completion (Hamill, 2003). In the same vein, as self-efficacy is regarded as one of the most significant issues for EFL learners to increase their resilience and buoyancy, it seems to be very significant for the educator to assist their learners to cultivate their self-efficacy.

## Takeaways for L2 Education

Teachers can heighten the level of learners’ self-efficacy through numerous possible teaching procedures. Also, self-efficacy can change, and so, can be increased in students to enhance their scholastic buoyancy and consequently academic buoyancy can keep them safe from everyday scholastic difficulties. This is beneficial knowledge to learners, educators, managers, and policymakers to make sure that students should try to strengthen their scholastic buoyancy and improve their capability of fighting small scholastic obstacles that might impede their attainment of objectives. In addition, strengthening self-efficacy in students could improve academic buoyancy; therefore, this allows students to fight difficulties inside the educational setting and attain scholastic objectives that might result in elevated economic growth.

A greater degree of self-efficacy formulae the foundation of a low level of apprehension and great degrees of commitment that consequently leads to more buoyancy. Accordingly, parents are supposed to reinforce their association with their children to develop and boost their self-efficacy which sequentially develops their educational achievement. Moreover, social support has been projected as a source to efficiently regulate and control anxiety and work effectively among learners through its association to generate from numerous experiential trials, like trials on trauma, apprehension, and efficacy (Greenberger et al., 2000; Dziegielewski et al., 2004). Societal support makes an individual feel cared for, and their existence makes a difference in the lives of others. Moreover, compassion and societal connections are fundamental human needs. For learners, the presence of this support framework assists with building resilience, specifically when encountering college life difficulties. When highly resilient individuals encounter issues or distressing occurrences, they will embrace the circumstance and stand up again to figure out a solution. They are capable of working well and coping with distressing circumstances.

It is similarly proposed that to boost resilience and buoyancy in learners, teacher trainers should train their educators to be familiar with some strategies to be employed in their classes and provide significant prospects for communication in the classroom, improve and encourage their societal support, stimulate constructive peer relationships by tasks presented in the class, nurture their self-assurance and stimulate learners’ self-efficacy. The outcome can function as source materials for scholars, experts, and university specialists who are fascinated about inspecting problems in psychology like resiliency and social support. Consequently, more studies can be done as a contribution to a review of the literature.

The current review presents some evidence that self-efficacy and social support may be connected to learners’ resilience and buoyancy, consequently, it maintains further investigations to study these two aspects for both educators and students. Future scholars are stimulated to conduct more experimental types of research regarding the mutual relations among the variables of this study to locate these results in EFL settings (Xue, 2021; Liu and Chu, 2022; Wang et al., 2022).

## Data Availability Statement

The original contributions presented in the study are included in the article/supplementary material, further inquiries can be directed to the corresponding author/s.

## Ethics Statement

The studies involving human participants were reviewed and approved by Southwestern University of Finance and Economics Academic Ethics Committee. The patients/participants provided their written informed consent to participate in this study.

## Author Contributions

The author confirms being the sole contributor of this work and has approved it for publication.

## Conflict of Interest

The author declares that the research was conducted in the absence of any commercial or financial relationships that could be construed as a potential conflict of interest.

## Publisher’s Note

All claims expressed in this article are solely those of the authors and do not necessarily represent those of their affiliated organizations, or those of the publisher, the editors and the reviewers. Any product that may be evaluated in this article, or claim that may be made by its manufacturer, is not guaranteed or endorsed by the publisher.
